# Weight loss and weight gain among participants in a community-based weight loss Challenge

**DOI:** 10.1186/s40608-018-0225-1

**Published:** 2019-01-04

**Authors:** Merrill D. Funk, MinJae Lee, Michelle L. Vidoni, Belinda M. Reininger

**Affiliations:** 1 Department of Health and Human Performance, The University of Texas Rio Grande Valley, Brownsville, TX USA; 2 Department of Kinesiology and Outdoor Recreation, Southern Utah University, 351 W. University Blvd, Cedar City, UT 84720 USA; 3 McGovern Medical School, The University of Texas Health Science Center at Houston, Houston, TX USA; 4 The University of Texas School of Public Health, Health Science Center at Houston, Houston, TX USA; 5 The University of Texas School of Public Health, Health Science Center at Houston, Brownsville Regional Campus, Brownsville, TX USA

**Keywords:** Weight loss, Weight maintenance, Community health, Weight management program

## Abstract

**Background:**

To describe the characteristics of participants who registered for multiple annual offerings of a community-based weight loss program called The Challenge, and to determine participant characteristics associated with weight change over multiple offerings of The Challenge occurring during the years 2010–2016.

**Methods:**

Multivariable linear mixed effects analyses were conducted to describe percent weight change within and between offerings of The Challenge by participant characteristics.

**Results:**

There were 669 and 575 participants included in the within and between analyses, respectively, for offerings of The Challenge. Among the 434 participants who lost weight in their first attempt at The Challenge and completed the initial weigh-in for a subsequent offering of The Challenge, 22.4% maintained their weight loss or had greater weight loss by the next Challenge, 40.3% gained back some weight, and 37.3% gained back all or more of the weight they lost during their first Challenge. Men had a significantly greater percent weight loss compared to women in their first and second Challenge and men were more likely to gain weight between Challenges. Participants who returned to more Challenges had a greater accumulated percent weight loss compared to those who returned to fewer Challenges.

**Conclusions:**

The current weight loss Challenge appears to contribute to helping a percentage of participants lose weight and maintain some or all of the weight loss.

## Background

Approximately 70% of the US population has been diagnosed with overweight and various levels of obesity, making weight loss and weight loss maintenance an important public health concern [1]. Obesity has recently been classified as a disease [2] and is also a risk factor for chronic health conditions and mortality [3]. Developing methods to prevent increases in obesity and managing excess weight in those currently struggling with obesity should therefore be a high public health priority. Weight loss is reported consistently during combined diet and exercise interventions and diet alone interventions, but as participants return to their normal lifestyle about half of the weight lost during the intervention is regained within a year [4, 5].

Several factors have been shown to contribute to losing weight and maintaining weight loss for individuals with obesity or overweight prior to weight loss, though recent research indicates that there may be unique factors influencing weight regain for individuals with extreme obesity prior to weight loss [6, 7]. A wide range of dietary strategies and diet compositions have been shown to be effective at producing weight loss as long as a caloric deficit is achieved [8, 9]. Caloric restriction has been shown to be the primary means of weight loss with physical activity as an important component to effectively maintain weight loss [10]. Social factors such as group exercise sessions or exercising with a friend play a larger role in weight loss for young adults while older adults are motivated more by health concerns [11]. Participants who successfully maintain weight loss report using behavioral strategies to control dietary calorie and fat intake, are physically active, and weigh themselves frequently [12–15]. Longer periods of successful weight loss maintenance (2–5 years) have been associated with decreased likelihood of weight regain [16, 17]. Additionally, people who are able to maintain weight loss longer use fewer strategies and less effort to lose and maintain weight loss [18].

The need for effective weight loss and weight loss maintenance strategies is particularly important in South Texas where the burden of obesity is higher than other parts of the U.S. Hispanic adults in the United States have documented higher rates of obesity compared to average rates in the United States (37.9% vs. 33.9% respectively) [19] with greater prevalence of overweight and obesity in children and adults in South Texas compared to the rest of the country [20]. A random sample of adults aged 35–64 years in Cameron County in South Texas included over 70% of participants who were born in Mexico and showed an obesity prevalence of greater than 55% [20]. This area also has high rates of type 2 diabetes and poor metabolic health [21]. Understanding characteristics of individuals who participate in weight loss and maintenance of weight loss in South Texas may lead to important improvements in health status in that region and provide insight to other regions with Hispanic populations.

There were two objectives for this study. The first aim was to determine participant characteristics associated with weight change during multiple offerings of a community-based weight loss program called The Challenge (offered once per year beginning in January and lasting approximately 14 weeks each year) and the second aim was to determine participant characteristics associated with weight change between multiple offerings of The Challenge. Operational terms used in this paper are defined as follows: weight loss – a participant having less weight at the conclusion of one annual Challenge compared to the start; weight gain – a participant having more weight at the conclusion of one annual Challenge compared to the start; weight maintenance – a participant having the same or less weight at the start of their second annual Challenge compared to the completion of their first Challenge; weight regain – a participant having more weight at the start of their second annual Challenge compared to the completion of their first Challenge. This study provides insight into weight loss and weight loss maintenance associated with a community-based weight loss program.

## Methods

A free community-based weight loss program has provided support for many people in the South Texas area to improve their physical activity and dietary habits. Since 2010, a 14-week community weight loss program called The Challenge has been offered every January and held annually in this region with the number of adult participants aged 18 and older with overweight or obesity growing from 400 participants during the first year to over 1200 participants each year for the last several years. Participants were allowed to enroll every year regardless of prior participation in The Challenge during a previous year. Unlike clinically-based weight loss programs, there was no diet or exercise routine specifically prescribed for the participants but rather broad guidelines were given along with free programs and resources to pick and choose which would directly support these lifestyle changes. During all years during which The Challenge has been offered, there was an active community wide campaign to promote physical activity and healthful food choices in the region. The messages of this campaign and The Challenge were purposefully aligned around meeting physical activity guidelines and the importance of fruit, vegetable, and water consumption [22–25]. Participants could choose to enroll as individuals or as a small self-selected group to provide social support. This community-based program may provide greater opportunity for population-level impact on obesity compared to a clinic-based program [26].

The Challenge was open to anyone living in or around the city where measurements were taken and were completely voluntary. Participants were at least 18 years of age, not pregnant, and free of any medical conditions for which weight loss would be harmful. Participants were only tracked through their registration at the beginning and end of each annual Challenge and therefore information regarding the status of participants who failed to complete a Challenge or who did not return to a second Challenge was unknown. At the beginning of each Challenge, participants arrived at a predetermined location (library, park, or workplace) to complete sign-up information and obtain baseline measures. All measures and consent forms were taken by trained staff members. Participants were connected to free programs and information throughout The Challenge which may have been slightly modified each year based on participant feedback and available resources. During each round of The Challenge participants were initially made aware of and then provided reminders of opportunities to support their weight loss journey depending on the resources available in the community at that time. For example, in the early years of The Challenge there were less than 30 free exercise classes available for participants to attend, but in the later years there were nearly 100 free exercise classes. Nutritional classes and on-line resources, including options for recording food and physical activity were promoted, but not required. Weight and blood pressure assessments were offered at many but not all classes. Text message reminders of these resources and motivational tips were provided three times per week. At the conclusion of each Challenge (14 weeks average length), a final weigh-in was offered. Participants were not required to attend, but encouraged. Approximately 22% of participants (range from 30 to 16%) who began an annual Challenge attended the final weigh-in. As well, prizes were given to individuals and groups with the greatest percentage of weight loss along with raffles and prizes for participation. The Institutional Review Board at University of Texas Health Science Center Houston (study number HSC-SPH-13-0531) approved this study.

Anthropometrics were collected by a staff team of two people. One team member operated the measurement apparatus while the other person recorded findings. Weight was measured using a calibrated electronic Tanita scale and height was measured using a self-standing stadiometer to the nearest 1/8 in.. Height and weight were used to calculate body mass index (BMI; kg/m^2^). Waist and hip measurements were taken twice using a standard plastic tape measure (no tensometer) in order to calculate the waist/hip ratio using the average of the two measures to the nearest 1/8 in.. Waist measurements were taken at the circumference around the umbilicus and hip measurements were measured at the largest part of the hips.

Weight change was defined in aim 1 as percent weight change during one Challenge. The percent weight change per Challenge was measured as ((initial weigh-in at first Challenge – final weigh-in at first Challenge)/(initial weigh-in at first Challenge) × 100%). Weight change in aim 2 was defined as weight change between the end of one annual Challenge and the start of a subsequent Challenge (about 38 weeks between consecutive Challenges or longer if they did not attend during consecutive years). The percent weight change between Challenges was measured as ((initial weigh-in at second Challenge – final weigh-in at first Challenge)/(initial weigh-in at second Challenge) × 100%).

Additional participant characteristics were collected on a registration form which was subsequently checked by a staff member: gender, ethnicity (Hispanic or white), language preference (Spanish or English), and participating category (individual, small group or large group). Age was also obtained and categorized as either above 40 or 40 years and below.

### Statistical analyses

Participants who registered for at least two offerings of The Challenge (could be non-consecutive years) with complete data for at least one Challenge, both weigh-ins, were included in aim 1. To be included into the analysis for aim 2, participants had to have a final weigh-in for a first Challenge and initial weigh-in for a second Challenge. Multivariable linear mixed effects models were conducted for both aims 1 and 2. Linear mixed effect models account for intra-participant correlations inherent in repeated measure study designs. Participant characteristics possibly associated with weight change including, gender, age at registration, language, participating category, initial weight, year gaps between Challenges and total number of Challenges, were examined. Potential interaction effects were also evaluated while developing the final multivariable models. Regardless of its significance, an interaction effect between intervention time (i.e., Challenge) and gender was explored for both aim 1 and 2 in order to show a gender effect at each Challenge separately. In aim 1, adjusted mean difference of percent weight change was estimated where the positive difference signifies weight loss and in aim 2, a positive adjusted mean difference of percent weight change indicates weight gain.

Further, among study participants who lost weight during their first Challenge, weight change between participants’ first and second Challenge was categorized to describe weight loss and regain. Weight loss maintenance or additional weight loss was defined as participants either maintaining weight loss obtained during their first Challenge or losing additional weight by the initial weigh-in of their second Challenge. Some weight regain was defined as participants having gained back a portion, but not all of the weight they lost during their first Challenge by the time of the initial weigh-in of their second Challenge. Total weight regain was defined as participants having gained back all of the lost weight during their first Challenge by the initial weigh-in for their second Challenge. All analyses were performed using SAS 9.4 (SAS Institute Inc., Cary, NC) at a statistical significance level of 0.05.

## Results

**Fig. 1:**
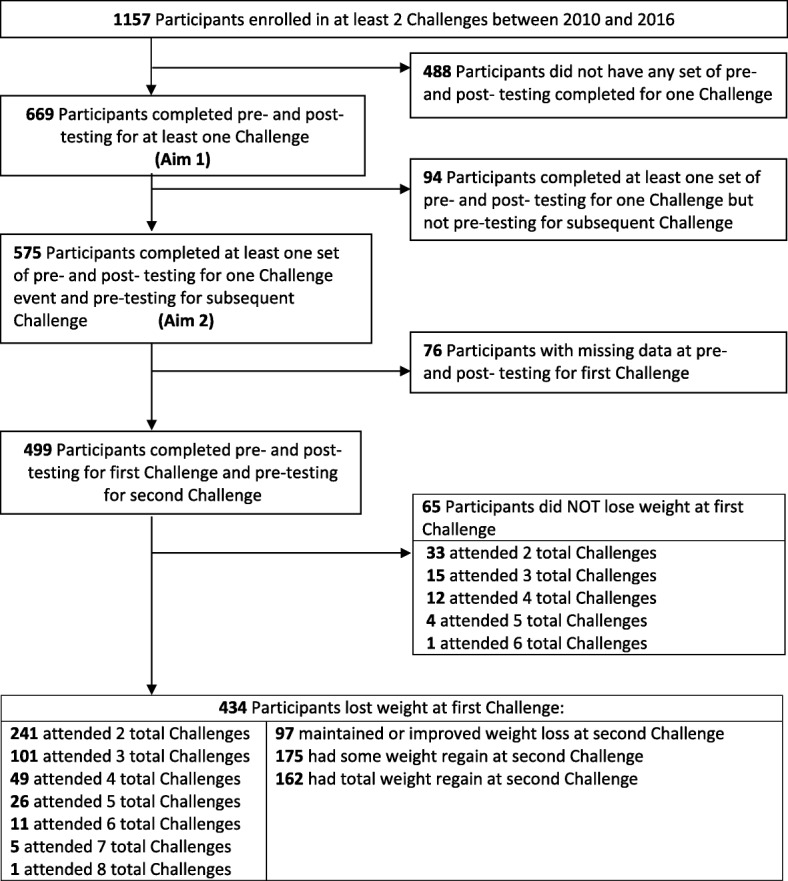
Participant flow diagram

Among the 1157 participants who enrolled in at least two Challenges between 2010 and 2016, 669 participants were included in the analysis for aim 1 and 575 participants were included in the analysis for aim 2 (see Fig. 1). Among the 499 participants who did not have any missing data, 434 (87.0%) lost weight during their first Challenge. Of the 434 who lost weight at the first Challenge, 241 returned to at least one additional Challenge. Frequencies for weight loss maintenance, some weight regain, and total weight regain from the conclusion of the first Challenge to the initiation of a second Challenge are also reported in Fig. 1. Among the 434 participants who lost weight, 97 (22.4%) maintained their weight loss or had even greater weight loss by the initial weigh-in of the next Challenge they attended, 175 (40.3%) gained back some, but not all of the weight they lost during their first Challenge, and 162 (37.3%) gained back all of the weight they lost during their first Challenge.

Presented in Table 1 are descriptive statistics of the study populations for each aim. Most participants were Hispanic (~ 90%). In both analysis groups, mean estimates for BMI (≥30 kg/m^2^), waist circumference (> 40 in. for men and > 35 in. for women), and waist to hip ratio (> 0.90 for men and > 0.85 for women) were above obesity cut offs.

**Table 1:** Descriptive characteristics for participants who were included in aim 1 (*N* = 669) and aim 2 (*N* = 575) at their first Challenge registration

	Aim 1, *N* = 669		Aim 2, *N* = 575	
Variable	Men (*n* = 154)	Women (*n* = 515)	Men (*n* = 131)	Women (*n* = 444)
Age > 40 years, n(%)	70 (45.5)	240 (46.6)	59 (45.0)	212 (47.8)
Hispanic, n(%)^a^	120 (87.6)	451 (95.6)	104 (86.7)	395 (95.8)
Spanish language, n(%)	16 (10.4)	116 (22.5)	14 (10.7)	95 (21.4)
BMI, kg/m^2^, mean (SD)	35.6 (6.8)	34.4 (7.2)	35.8 (7.1)	34.4 (7.1)
Waist circumference, inches, mean (SD)^b^	45.7 (6.0)	42.2 (9.6)	45.8 (6.2)	42.4 (10.0)
Waist to hip ratio, mean (SD)^b^	0.98 (0.06)	0.91 (0.14)	0.99 (0.06)	0.91 (0.15)
Weight, lbs., mean (SD)	235.1 (48.8)	189.9 (43.6)	236.2 (48.9)	190.3 (43.4)

^a^missing *n* = 60 for aim 1, *n* = 43 for aim 2

^b^missing *n* = 132 for aim 1, *n* = 116 for aim 2

### Aim one: Weight change during Challenges

**Fig. 2:**
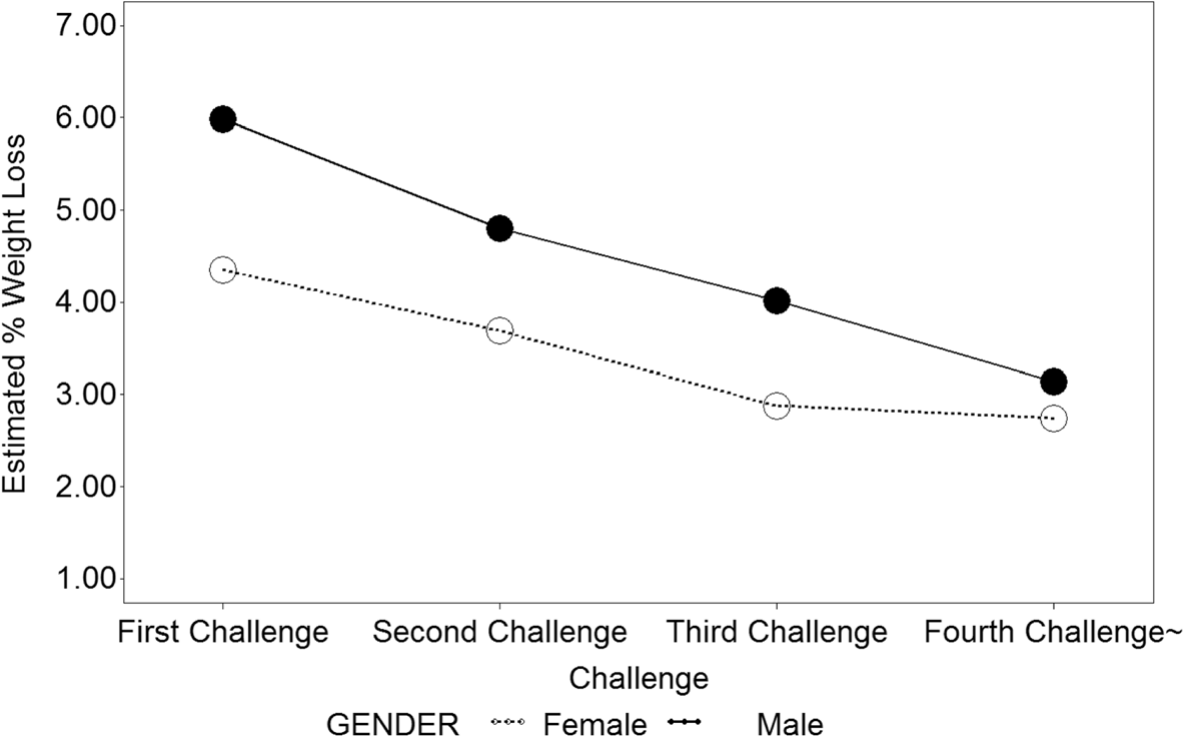
Estimated percent weight loss across Challenges by gender

Six hundred sixty-nine participants were included in this analysis because they completed initial and final weigh-ins for at least one Challenge they participated in from 2010 to 2016. As shown in Fig. 2 and Table 2 from the multivariable longitudinal model, men had a significantly greater adjusted mean difference of percent weight loss compared to women in their first and second Challenge (first Challenge: 1.63%, *p* = 0.0007; second Challenge: 1.12%, *p* = 0.0438); however, the significance disappeared after two Challenges, likely due to the decreasing number of participants attending three or more Challenges. Percent weight loss significantly decreased every time participants returned to each additional Challenge for both men and women. For example, compared to Challenge 1, adjusted mean difference was − 2.84% (*p* = 0.0016) for men and − 1.61% (*p* = 0.0029) for women at Challenge 4, while − 1.18% (*p* = 0.0384) for men and − 0.66% (*p* = 0.0291) for women at Challenge 2 (not shown in Table 2). We also found that those participants with higher initial weight for their first Challenge were significantly more likely to have a greater percent weight loss than those with a lower weight at initial weigh-in though this value may not have clinical significance (adjusted mean difference = 0.01%, *p* = < 0.0001). Participants who returned to more Challenges had a greater adjusted mean difference in accumulated percent weight loss across the years of their participation (adjusted mean difference = 0.27% per Challenge, *p* = 0.0407) compared to the cumulative effect of those who returned to fewer Challenges. There was no significant association of weight loss with language preference, participating category, or age in multivariable mixed effect models. Despite not being statistically significant, participants who signed up with a group were more likely to lose weight than those who signed up as individuals (adjusted mean difference = 0.01%, *p* = 0.959).

**Table 2:** Factors associated with longitudinal percent weight loss based on multivariable mixed effect model (*n* = 699)

Variable	Adjusted mean difference of % weight loss^a^ (95% CI)	*p*-value
Male vs. Female		
At Challenge 1	1.63 (0.70, 2.57)	0.0007
At Challenge 2	1.12 (0.03, 2.21)	0.0438
At Challenge 3	1.14 (−0.62, 2.90)	0.2048
At Challenge 4 & more	0.40 (−1.50, 2.30)	0.6774
Weight at first Challenge registration	0.01 (0.01, 0.02)	< 0.0001
Age at first Challenge	−0.02 (− 0.04, 0.01)	0.2650
English vs Spanish	−0.11 (− 0.84, 0.62)	0.7703
Group vs individual	0.01 (−0.55, 0.58)	0.9590
Number of repeated Challenges	0.27 (0.01, 0.52)	0.0407

^a^Positive estimate means more weight loss

% weight loss = ((initial weigh-in at first Challenge – final weigh-in at first Challenge)/(initial weigh-in at first Challenge) × 100%)

### Aim 2 analysis: Weight change between challenges

**Fig. 3:**
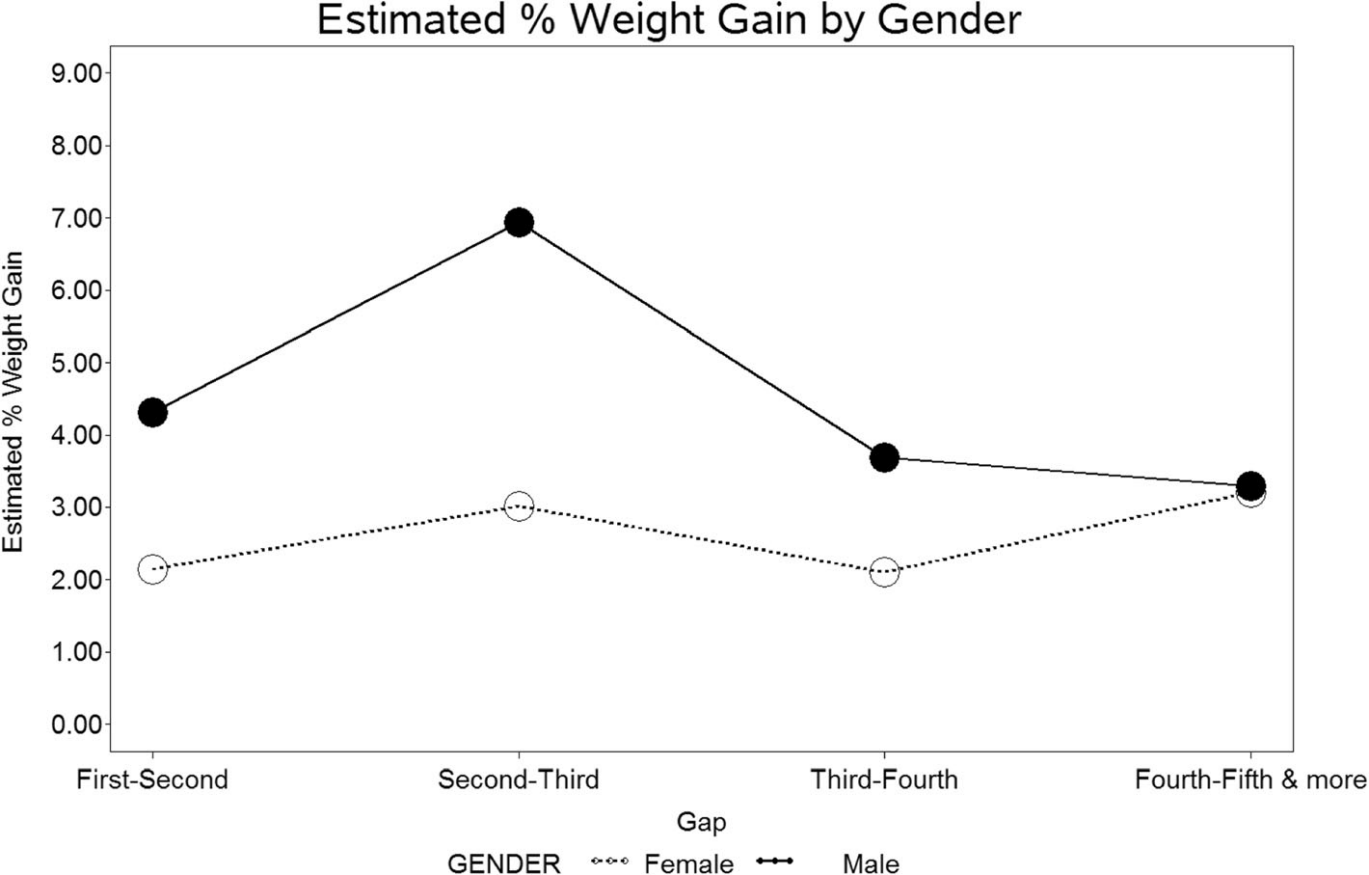
Estimated percent weight gain across Challenges by gender

Five hundred seventy-five participants were included in this analysis because they had weight data available for at least a final weigh-in for one Challenge and initial weigh-in data for a following Challenge. Based on the multivariable longitudinal model results shown in Fig. 3 and Table 3, males were more likely to have a positive weight change, i.e. weight gain, between Challenges compared to women (adjusted mean difference between first and second Challenges: 2.17%, *p* = 0.0037; between second and third Challenges: 3.93%, *p* = 0.0126). There was no statistically significant difference in weight gain found by gender in subsequent Challenges though the number of participants attending more than three Challenges was small. Greater weight loss at the first Challenge was significantly associated with more weight gain (adjusted mean difference = 2.29%, *p* = 0.0020). Also, those participants with a higher weight at the end of the previous Challenge were significantly more likely to have less percent weight gain at a subsequent Challenge (adjusted mean difference = − 0.02%, *p* = 0.0014). Those participants who competed in a group category were significantly more likely to have weight gain at subsequent Challenges compared to those who competed as an individual (adjusted mean difference = 1.40%, *p* = 0.0107). More years between Challenges was associated with greater percent weight gain (adjusted mean difference = 0.80, *p* = 0.0124). There was no significant association of weight gain with language preference or age in multivariable mixed effect models.

**Table 3:** Factors associated with longitudinal percent weight gain based on multivariable mixed effect model (*n* = 575)

Variable	Adjusted mean difference of % weight regain^a^ (95% CI)	*p*-value
Male vs. Female		
At Challenge 1–2	2.17 (0.71, 3.62)	0.0037
At Challenge 2–3	3.93 (0.85, 7.00)	0.0126
At Challenge 3–4	1.58 (−2.44, 5.60)	0.4390
At Challenge 4–5 & more	0.08 (−4.24, 4.40)	0.9695
% weight loss for first Challenge	2.29 (0.85, 3.72)	0.0020
Previous Challenge final weight	−0.02 (− 0.03, − 0.01)	0.0014
Age at first Challenge	− 0.04 (− 0.09, 0.00)	0.0651
English vs. Spanish	− 0.20 (−1.48, 1.08)	0.7591
Group vs. Individual	1.40 (0.33, 2.48)	0.0107
Year gap between Challenges	0.80 (0.18, 1.43)	0.0124

^a^Positive estimates means more weight gain

% weight gain = ((initial weigh-in at second Challenge – final weigh-in at first Challenge)/(initial weigh-in at second Challenge) × 100%)

## Discussion

This study examined weight change over time among community members participating in a free community-based weight loss program. Few studies have examined community samples, but rather most weight loss studies examine participants from clinically driven weight loss programs [4, 5, 9, 27]. During any given Challenge enrollment, about 65% of participants lost weight, which may be lower than the percentage of participants losing weight in a structured clinical weight loss program. Efficacy trials which are tested under ideal conditions in a randomized controlled trail format “tend to have better outcomes than effectiveness trials” which are performed in real-world conditions [26]. Most research on clinical and commercial weight loss programs report mean values for weight loss for the program and it is not usually stated what percentage of participants lose weight. This may be because generally all but a few participants lose weight in a highly structured setting with high adherence and participants are dropped from the study if they do not follow the designed program. In a community setting, physiological, psychological, and environmental factors interact to produce larger heterogeneity in weight loss outcomes [28]. Community-based weight loss programs can play an important role in disseminating principles learned from highly structured clinical programs to larger populations [26].

This study found that gender and number of repeated Challenges for both males and females were significantly associated with greater percent weight loss. Males likely lost more weight than females initially due to differences in percentage of muscle mass, hormones, and other physiological characteristics [29]. A systematic review on gender differences in weight loss interventions found that men lose more weight than women, but women are better with weight loss maintenance possibly due to a slower initial weight loss [30]. Because participants were not obligated to return for final weigh-in assessments at the conclusion at each Challenge or to return to repeated Challenges, those who did return were likely those who found success with prior attempts at weight loss through this program while those who did not return and were lost to follow-up likely did not experience success with The Challenge. Initial weight loss goals for those trying to lose weight often exceed what can reasonably be expected and this may cause them not to return to the final weigh-in and may prevent them from participating in additional Challenges if they do not obtain the success they think they should obtain [31]. Therefore, those who attended more Challenges may have been able to obtain a greater percent weight loss over time, though the amount of weight loss decreased for each additional Challenge for those who participated in multiple Challenges.

Some participants struggled to maintain their weight loss thus we also examined factors associated with weight change between Challenges. We found that males and females who initially lost a greater percent body weight were more likely to gain weight. However, we also found in the second analysis that those participants with a higher weight at the final weigh-in of a previous Challenge were likely to have a lower mean increase in their weight when they returned to a later Challenge. A meta-analysis reported that participants in structured weight loss programs, who lost a significant amount of weight, were only able to maintain a sustained reduction of 3–6% of their initial body weight at 48 months follow-up [5]. Among 434 participants in this program who lost weight during their first Challenge (an average of 5.45% weight lost), 22.4% of the participants who lost weight during their first Challenge were able to maintain or have greater weight loss (an average of 3.49% weight kept off) by the initial weigh-in for their second Challenge.

Somewhat surprising, while we found no differences for participants who competed as an individual versus in a group in percent weight loss, we did find that participants who competed in a Challenge as part of a group were more likely to gain weight between Challenges. Past literature shows the importance of social support in weight loss especially in younger people with both positive and negative effects [18, 32] however, perhaps we are seeing the impact of lack of group support between Challenges resulting in greater weight gain compared to individuals whose level of support was more constant because they competed as an individual. Not surprising, we also found that those who waited a longer amount of time to participate in a subsequent Challenge had greater weight gain. Diet and exercise strategies maintained following weight loss will greatly determine the extent to which the weight loss is maintained [33]. When a Challenge ends and the incentive and support to maintain the strategies used to lose weight are removed [12–15], the physiological drive to return to a person’s original weight and return to their old patterns of eating and physical activity make weight regain common [34].

Community-based weight loss programs have the potential to affect large populations and potentially change overall population health. A combined report from the American College of Cardiology, American Heart Association, and The Obesity Society suggests evidence-based practices that should be considered when prescribing a weight loss program, though means to apply these principles should be adapted to a specific population [35]. A program developed for low income Latinos modeled the Diabetes Prevention Program and evaluated the effectiveness of adding community health workers compared to standard care over a 24 month weight loss program [36]. Participants in the intervention group lost more weight than the control group at 6 months, but differences between groups were lost at 12 and 24 months as the intervention group received less intensive intervention. Men in the intervention group lost more weight than women at all time points similar to the findings in the current study. It appears that a common difficulty with community-based interventions is that when intervention efforts diminish, so do the results achieved through the intervention.

Based on the current findings, large-scale community weight loss programs should seek ways to establish and maintain contact with participants with incentives built into the program to keep them engaged to effectively lose weight and return to the final weigh-in and to maintain weight loss long-term. Maintenance of social support along with teaching lifestyle skills within the context of the local environment will likely help improve effectiveness of community-based weight loss programs.

## Conclusion

Participants who lost more weight came back for more annual weight loss Challenges, but experienced diminishing weight loss each time they returned with men losing more weight than women. The current Challenge appears to be successful in helping a percentage of participants to lose weight and reduce weight gain if they return for another Challenge. This potentially means that there are lifestyle changes occurring to help these positive changes persist during the intermission between Challenges.

## Abbreviation

BMI: Body mass index
